# Kinetics of the neutralising antibody response in patients with hand, foot, and mouth disease caused by EV-A71: A longitudinal cohort study in Zhengzhou during 2017-2019

**DOI:** 10.1016/j.ebiom.2021.103398

**Published:** 2021-05-25

**Authors:** Qi Qiu, Jiaxin Zhou, Yibing Cheng, Yonghong Zhou, Lu Liang, Peng Cui, Yingying Xue, Lili Wang, Kai Wang, Haijun Wang, Peng Li, Junbo Chen, Yu Li, Lance Turtle, Hongjie Yu

**Affiliations:** a School of Public Health, Fudan University, Key Laboratory of Public Health Safety, Ministry of Education, Shanghai, China; b Hospital Affiliated to Zhengzhou University, Henan Children's Hospital, Zhengzhou, China; c West China School of Public Health and West China Fourth Hospital, Sichuan University, Chengdu, China; d Division of Infectious Disease, Key Laboratory of Surveillance and Early-warning on Infectious Disease, Chinese Centre for Disease Control and Prevention, Beijing, China; e WHO Collaborating Centre for Infectious Disease Epidemiology and Control, School of Public Health, Li Ka Shing Faculty of Medicine, The University of Hong Kong, Hong Kong Special Administrative Region, China; f NIHR Health Protection Research Unit for Emerging and Zoonotic Infections, University of Liverpool, Liverpool, United Kingdom; g Tropical & Infectious Disease Unit, Royal Liverpool University Hospital (member of Liverpool Health Partners), Liverpool, United Kingdom; h Department of infectious diseases, Huashan Hospital, Fudan University, Shanghai, China

**Keywords:** HFMD, EV-A71, Neutralising antibody, Acute phase, Convalescent phase

## Abstract

**Background:**

Hand, foot, and mouth disease (HFMD) caused by enterovirus A71 (EV-A71) poses a serious threat to children's health. Kinetics of the neutralising antibody (NAb) response in EV-A71 infected HFMD patients remains unclear. The ideal sampling time of paired serum samples for serological diagnosis of EV-A71 infection is not well defined.

**Methods:**

HFMD inpatients admitted to Henan Children's Hospital between February 15, 2017 and February 15, 2018 were enrolled. Serial serum samples collected during hospitalisation and up to 1.5 years after discharge were tested for NAb against EV-A71. Random intercept modelling with B-spline was conducted to characterize the kinetics of the EV-A71 NAb response over time after illness onset.

**Findings:**

A total of 524 serum samples from 264 EV-A71 RNA positive HFMD inpatients were collected. NAb titres of EV-A71 infected patients were estimated to increase from 40 (95% CI: 9-180) at the day of onset to the peak of 2417 (95% CI: 1859-3143) at day 13, then remained above 1240 until 26 months. For serological diagnosis of EV-A71 infection, if at least a 4-fold rise in titre was used as the criteria, the acute phase serum should be collected at 0-4 days, the corresponding convalescent serum should be collected 14.9 days (95% CI: 9.1-23.8) after illness onset.

**Interpretation:**

EV-A71 infection induced a strong and persistent humoral immune response in HFMD patients. The findings provide a scientific support for determining the collection time of paired serum samples for serological diagnosis of EV-A71 infected HFMD patients.

**Funding:**

National Science Fund for Distinguished Young Scholars

**Research in Context**

**Evidence before this study**

We searched PubMed for articles on antibody response against enterovirus A71 (EV-A71) published before March 15, 2020, with the search terms “EV71”, “EV-A71”, “enterovirus 71”, “Enterovirus A71”, “hand, foot, and mouth disease”, “HFMD”, “antibody response”, and “immune response” without language restrictions. Few studies have previously described the kinetics of EV-A71 NAb response in HFMD patients, which reported that the antibody response has already initiated on the day of illness onset, and the NAb titre increased with time within a few days. A recent study showed that the positive rate (60% *vs.* 100%) and GMTs (37.7 *vs.* 295.1) of EV-A71 neutralising antibody in the recovery period serum of HFMD patients increased significantly compared with the acute period. Paired sera for serological researches were empirically collected within one week and two weeks after illness onset, respectively, but lacked support from experimental evidence. To our knowledge, our study represents the first attempt to construct a kinetic model of the NAb response to EV-A71 over time in HFMD patients using data from serum samples at multiple time points during 2 years after illness onset.

**Added value of this study**

In this study, we described the kinetics of the EV-A71 NAb response during hospitalisation and for up to 26 months after recovery by using the data from a prospective cohort of EV-A71 infected HFMD inpatients. We found that the antibody response has already initiated once clinical symptoms appeared, NAb titre quickly peaked within two weeks after illness onset, and then remained at a high level until two years. For serological diagnosis of EV-A71 infection in HFMD patients, if a 4-fold rise was used as the criteria, the acute phase serum should be collected at 0-4 days, and the corresponding convalescent serum should be collected 15 days after illness onset. Our study provided a basis for understanding host-pathogen interactions of EV-A71 infection and informing the serological diagnosis of HFMD caused by EV-A71.

**Implications of all the available evidence**

EV-A71 infection induced a strong and persistent humoral immune response in patients with HFMD. The benefit of IVIG for the treatment of HFMD should be questioned as strong and persistent NAb responses were elicited by EV-A71 infection. For serological diagnosis of EV-A71 infection in HFMD patients, the acute phase sample was recommended to be taken as early as possible, preferably within 3-4 days after illness onset. The corresponding convalescent serum should be collected 2 weeks after illness onset.

Alt-text: Unlabelled box

## 1 Introduction

Hand, foot, and mouth disease (HFMD) is a common disease caused by enteroviruses, posing a serious threat to children's health, especially in China [1]. Most cases of HFMD are mild and self-limiting, but some cases, mainly caused by enterovirus A71 (EV-A71), may be severe and develop neurological and cardiopulmonary complications, resulting in long-term sequelae, or even death [2]. EV-A71 is also responsible for outbreaks and epidemics of HFMD, with EV-A71 C4a being the major genetic lineage circulating in mainland China in the past decade [3,4]. Moreover, in USA and Europe, EV-A71 has also been identified as the cause of outbreaks of neurological disease [5,6].

Neutralising antibody (NAb) plays a crucial part in protection from viral infection, and is one of the most important indicators of humoral immunity. The kinetics and duration of the NAb response in patients with HFMD caused by EV-A71 is not well understood. Serological diagnosis of an acute enterovirus infection classically relies on a 4-fold rise in the specific NAb between acute and recovery phases [7]. Due to widespread exposure to related viruses, NAb titres are often present early in illness, limiting the usefulness of this test, especially with increasing age [8]. However, there has been no clear definition of sampling time for paired serum samples in order to maximise usefulness in previous studies. Studying the pattern of the NAb response against EV-A71 is essential for understanding host-pathogen interactions and informing the serological diagnosis of HFMD caused by EV-A71.

In this study, we conducted a prospective cohort study in HFMD patients hospitalised in Henan Children's Hospital, and aimed to clarify the kinetics of the NAb response in EV-A71 infected HFMD patients during hospitalisation and up to 1.5 years after discharge, and to determine the optimal sampling time of paired samples for serological diagnosis of EV-A71 infection.

## 2 Methods

### 2.1 Participant enrolment and sample collection

Clinically diagnosed HFMD patients were recruited from department of infectious diseases and paediatric Intensive Care Unit (ICU) of Henan Children's Hospital between February 15, 2017 and February 15, 2018 [9]. Severe cases were defined as those meeting any of the following four criteria: (1) With additional central nervous system (CNS) complications such as encephalitis, brainstem encephalitis, encephalomyelitis, acute flaccid paralysis, meningitis, and other severe CNS syndromic presentations [10]; (2) Requirement for special treatment, including systematic corticosteroids or intravenous immunoglobulin (IVIG); (3) ICU admission during hospitalisation; (4) The length of hospital stay (LOS) over 5 days.

Among those enrolled during hospitalisation, a subgroup of patients was invited to attend for follow-up at 2 weeks, 3 months, 6 months and 1.5 years after discharge. Patients with the following conditions were excluded from follow-up: (1) premature children (born before 37 weeks); (2) any prior chronic respiratory, cardiac or other illness (e.g. congenital hypothyroidism, congenital epilepsy, asthma); (3) previous PICU admission or ventilation including during neonatal period; (4) prior learning disability or neurological regression; (5) prior delayed development or neurodevelopment. Data, including demographic and clinical information, were extracted from medical records using a standardized case record form.

Throat swabs were collected from participants within 48 hours after admission as part of routine care, and stool samples were also collected if available. Serum samples were collected during hospitalisation (at admission, disease progression, and/or discharge), as well as every follow-up visit after discharge. All samples were stored at -80 °C until testing. Diagnosis of enterovirus infection in the enrolled HFMD patients was as described previously [9]. Briefly, throat swabs were subjected to real-time RT-PCR and several nested RT-PCRs. For patients with negative throat swabs for enteroviruses, real-time RT-PCR using a commercial kit (Mole Bioscience; Taizhou, China) was performed on stool samples, when available, to supplement the diagnosis. EV-A71 RNA positive HFMD patients with at least one serum sample collected were the subjects of this study.

### 2.2 Neutralisation test

Virus neutralisation tests were conducted using the EV-A71 FY1708 strain (GenBank accession number: EU703812, genotype C4a), which was isolated from a patient during the 2008 HFMD outbreak in Fuyang city, Anhui province [3]. Serum samples were inactivated at 56°C for 30 min, serially diluted 2-fold (1:8 to 1:4096) in duplicate and incubated with 50 μl 100 TCID50 of EV-A71. After incubation at 37 °C for 2 h, the mixtures were added with human rhabdomyosarcoma cells in flat bottomed 96 well plates (1 × 10^5^ cells/ml) and incubated at 37 °C for 5-7 days. Cytopathic effect was observed by microscopy and measured by crystal violet staining. Each reaction plate included a positive antibody control (a polyclonal antibody generated by immunizing rabbits with a purified EV-A71 C4a intact virion, working concentration of 1:1024, from Sinovac Biotech Co., Ltd.), virus control, serum toxicity control, and cell control. A virus back titration was performed in each batch of test to determine the amount of virus was within the range of 32-320 TCID50/50μl. EV-A71 NAb standards (strongly positive, weakly positive and negative) from National Institutes for Food and Drug Control were used for quality control [11].

Antibody titres were defined as the reciprocal of the highest dilution capable of inhibiting 50% of the cytopathic effect and calculated by use of the Karber method [12]. For serum samples with NAb titres greater than 4096, assays were repeated with dilution of 1:8 to 1:16384. Titres below 8 and more than 16384 were assigned the value of 6 and 23170, respectively. A titre ≥32 was considered as seropositive.

### 2.3 Statistical analysis

NAb titres were log_2_ transformed before analysis. Geometric mean titre (GMT) and 95% confidence intervals (CI) were plotted at each time point after illness onset, and compared adjacent groups using Wilcoxon rank sum test, except for patients who have been previously vaccinated against EV-A71. Categorical variables were compared using the chi-square test or Fisher's exact test. A random intercept model with B-spline was established to simulate the average titre and 95% CI of EV-A71 NAb response over time in HFMD patients after illness onset, which was also known as the generalised linear mixed model (GLM) assuming that the antibody titre shows a curve of steady increase in the short term after onset. The knot and degree of B-spline in the model were selected based on Akaike information criterion. We pooled serological data from patients with at least two serum samples, and excluded patients who were still seronegative 2 months after illness onset or previously inoculated with EV-A71 vaccine from the model. Data from patients with only one serum sample were added in the model for a sensitivity analysis.

We fitted three parametric distributions (Weibull, gamma, and lognormal) with maximum-likelihood estimation of time-to-event data to estimate key parameters of disease course, medical treatment process and time for NAb increase of EV-A71 infected HFMD patients, which were used to define the acute phase of the disease and for the feasibility of clinical sampling. Illness onset was considered as the time when typical symptoms of HFMD such as rash or fever appeared in the early stage, that was day 0. The acute phase was defined as the time period from illness onset to the typical symptoms subsided. We did an average imputation with the GLM mentioned above and shortest distance decision filling for the missing data in acute phase based on the changing trend of GMT both at the population and individual level. Two other methods were used for sensitivity analysis, one was to calculate the GMTs directly, the other was to predict the GMT during the acute phase using a linear mixed model, which assuming that the antibody titre showed a linear increase in the short term after onset. Time intervals required for a 2, 4 and 8-fold increase in NAb titre were calculated to estimate the sampling time of convalescent serum corresponding to serum collected on each day of the acute phase. To minimize the effect of unbalanced sampling times during the follow-up period, we fitted the parametric distribution with interval and right censoring, and used 2 months as the final event time to reduce the effect of a large proportion of right censoring during analysis. The bootstrap method was used to estimate the sampling time of convalescent serum and its 95% CI.

All analyses were performed in R and SAS. P values of <0.05 were considered statistically significant.

### 2.4 Ethics

The study protocol and informed consent were reviewed and approved by the Institutional Review Boards of Henan Children's Hospital (IRB#YZ-17-006), Chinese Centre for Disease Control and Prevention (IRB#201624), and Public Health School of Fudan university (IRB#2017-12-0654). Written informed consent was obtained from parents and legal guardians of study participants on enrolment.

### 2.5 Role of funding source

The funders had no role in the study design, data collection, data analysis, data interpretation, or writing of the manuscript. The corresponding author had full access to all the data and had the final responsibility for the decision to submit for publication.

## 3 Results

### 3.1 Participants and serum samples

**Fig. 1:**
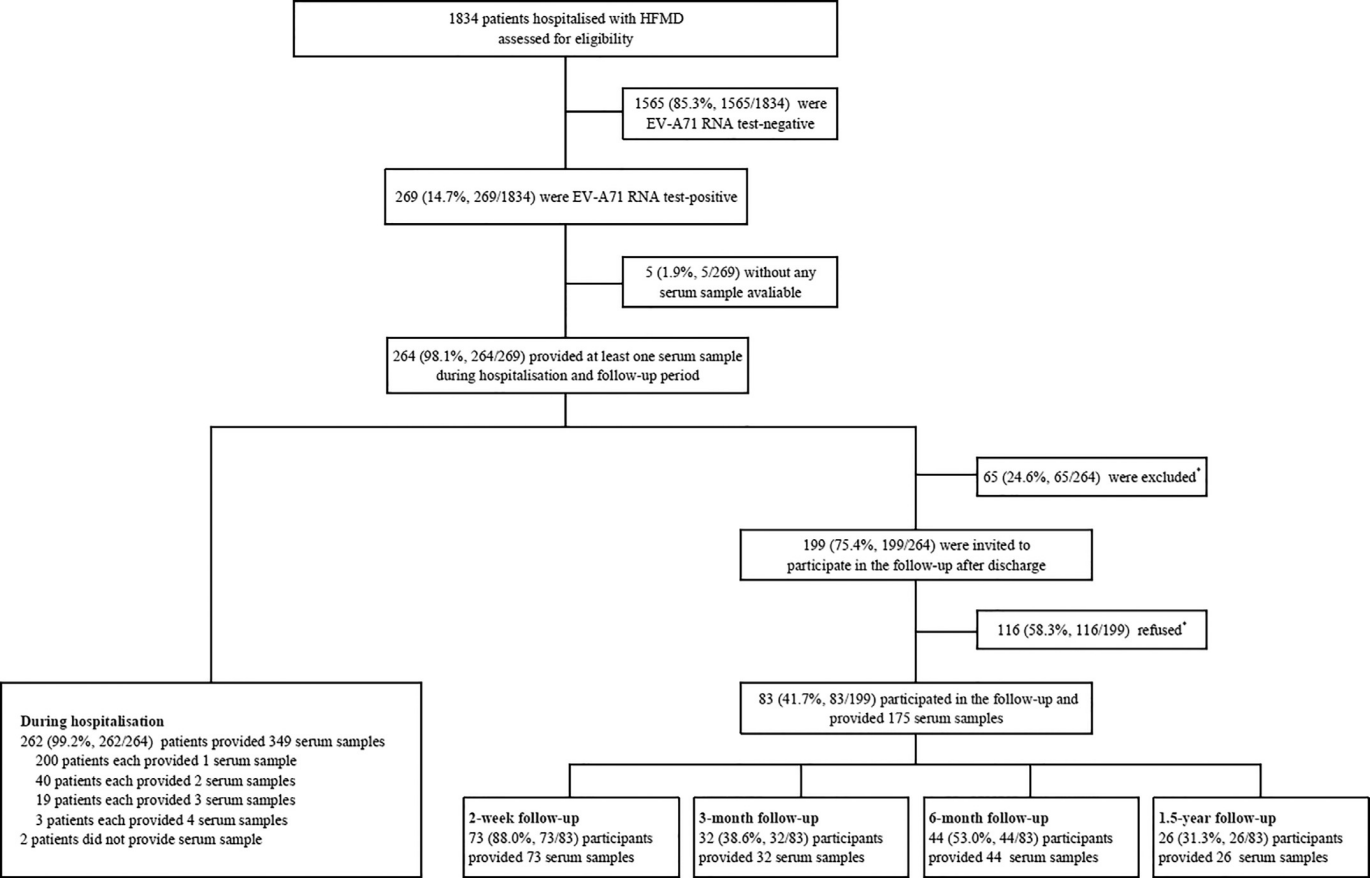
Flow chart of participants enrolment and serum samples collection throughout the study. 264 EV-A71 RNA positive patients were enrolled, and 83 (31.4%) of them participated in the follow-up after discharge.* Reasons for patients being excluded or refused to participant the follow-up were detailed in Supplementary Fig. 1.

From February 15, 2017 to February 15, 2018, a total of 1840 patients hospitalised with HFMD were enrolled into the cohort. According to the results of real-time RT-PCR and nested RT-PCRs, 264 (14.3%) patients were EV-A71 RNA positive, and 83 (31.4%) of them participated in the follow-up after discharge. Of these, 73 (88.0%), 32 (38.6%), 44 (53.0%) and 26 (31.3%) patients participated in the 2-week, 3-month, 6-month and 1.5-year follow-up, respectively (Fig. 1, Supplementary Fig. 1). A total of 524 serum samples were collected from 264 patients throughout, of which 349 sera were collected from 262 patients during hospitalisation, and 175 sera were collected from 83 patients during follow-up after discharge (Fig. 1, Supplementary Table 1). Each patient provided one to seven serum samples, and 114 (43.2%) of them provided at least two samples, 31 of these during hospitalisation, 81 of them during and after hospitalisation at follow up, and 2 only at follow up. The longest sampling time was 26 months after illness onset (Supplementary Table 2, Supplementary Fig.2).

### 3.2 Characteristics of participants

Demographic and clinical features of the patients were shown in Table 1. For 264 participants in the study, 163 (61.7%) of them were male. The age of the patients ranged from 2 months to 12 years, and 115 (43.6%) of them were under two years old. 4.5% (12/264) of the patients had been vaccinated against EV-A71 8 to 205 days before illness onset. We found no significant difference between vaccinated and unvaccinated patients regarding baseline characteristics (Supplementary Table 3). The median time from illness onset to hospital admission was 3 days (IQR: 2-4) after symptoms appeared, and the median length of stay was 5 days (IQR:4-6). A total of 143 (54.2%) patients were defined as severe cases. Compared with patients who were not invited or refused to participate in the follow up, severe cases account for the majority of patients who were invited (p=0.001) and participated in the follow-up (p=0.058) (Supplementary Table 4). One case, a 9-month-old girl with cardiopulmonary failure and encephalomyelitis, died at day 5 after illness onset during hospitalisation.

**Table 1:** Baseline characteristics of the patients who have provided serum samples during hospitalisation and after discharge.

Characteristics	All patients (N=264)	Provided serum samples during hospitalisation (n=262)	Provided serum samples after discharge (n=83)
Sex			
Male	163 (61.7)	161 (61.5)	52 (62.7)
Female	101 (38.3)	101 (38.5)	31 (37.3)
Age, years			
< 2	115 (43.6)	115 (43.9)	38 (45.8)
≥ 2	149 (56.4)	147 (56.1)	45 (54.2)
EV-A71 vaccination			
Yes	12 (4.5)	12 (4.6)	6 (7.2)
No	252 (95.5)	250 (95.4)	77 (92.8)
Median (IQR) time (days) from symptom onset to hospital admission	3 (2, 4)	3 (2, 4)	3 (2, 4)
Median (IQR) time (days) of LOS	5 (4, 6)	5 (4, 6)	5 (4, 6)
Clinical severity			
Mild	121 (45.8)	120 (45.8)	26 (31.3)
Severe	143 (54.2)	142 (54.2)	57 (68.7)

Data were no. (%) unless otherwise indicated. IQR, interquartile range; LOS, length of hospital stay.

### 3.3 NAb response after illness onset

EV-A71 NAb titres rapidly increased over time since illness onset (Supplementary Fig.3). Of patients who provided serum samples within one day of illness onset, 73.3% (11/15) had a NAb titre ≥32. For 262 patients with serum samples collected during hospitalisation, 181 (69.1%) developed a NAb titre ≥512 at a median of 4 days (IQR:3-6), and 31 (11.8%) developed a NAb titre ≥4096 at a median of 7 days (IQR: 4-10). NAb titres of two patients reached 23170 during hospitalisation at day 7 and 11, respectively. For the only fatal case, EV-A71 NAb titres reached 2048 four days after illness onset. The median sampling time of 150 patients with only one serum sample collected was 4 days after illness onset, with a median NAb titre of 724. 109 of the 264 patients had serial serum samples collected within 2 months after illness onset, 45.9% (50/109) of them had a 4-fold or more rise in NAb titres. The median sampling time of the first serum sample was 3 days (IQR: 2-4) after illness onset, and the median NAb titre was 362 (IQR: 181-949) in this group. In contrast, the median sampling time of the first serum sample of the remaining 54.1% (59/109) patients, who showed a less than 4-fold rise in NAb titre, was 4 days (IQR: 3-6) after illness onset, with a median NAb titre of 1448 (IQR: 362-2048). In addition, 14 patients remained seronegative until 2 months after the onset.

**Fig. 2:**
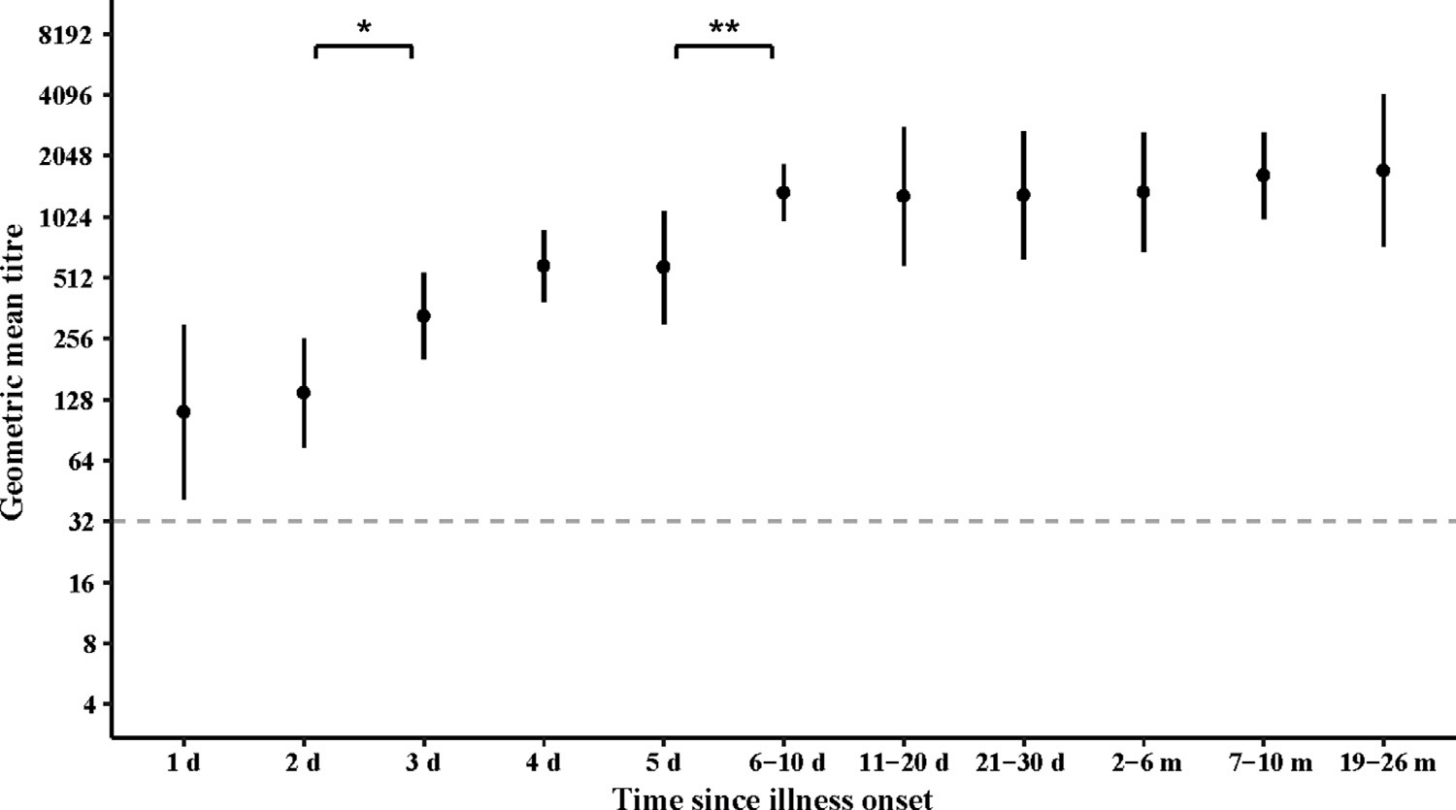
Geometric mean neutralising antibody titres (GMTs) at different times after illness onset among 493 samples collected from 252 unvaccinated patients. Serum samples were grouped by sampling time (1 day, 2 days, 3 days, 4 days, 5 days, 6-10 days, 11-20 days, 21-30 days, 2-6 months, 6-8 months, 7-10 months, and 19-26 months after illness onset). GMTs between adjacent groups were compared using Wilcoxon rank sum test.* indicates p<0.05, ^⁎⁎^ indicates p<0.01. Gray dotted line indicates threshold for positive titre (≥32).

**Fig. 3:**
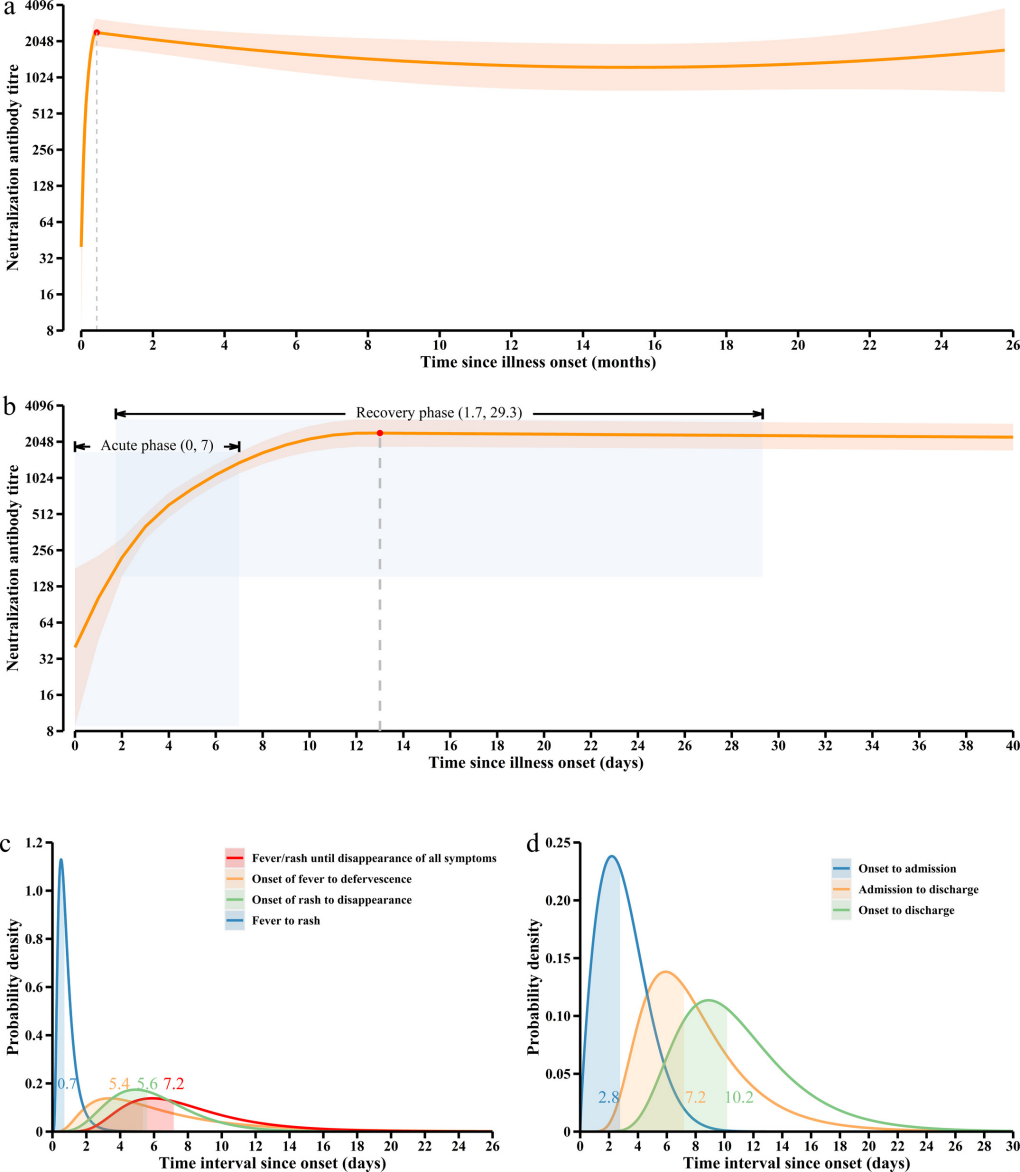
Model of EV-A71 neutralising antibody responses over time. A total of 310 serum samples from 92 unvaccinated patients who provided serial samples throughout were included. (a) average curve covers the whole study period (0-26 months after illness onset); (b) average curve between 0 and 40 days after illness onset; orange lines: average NAb antibody curve; orange ribbons: 95% CI around average antibody curve; light grey zones: when 4-fold rise was used as the standard, sampling time and titre range of serum samples in acute and convalescent phases; (c) time intervals relate to disease course of HFMD; (d) time intervals relate to medical treatment process.

Except for 12 patients who have been vaccinated against EV-A71 (Supplementary Fig. 4), NAb GMTs of the 252 unvaccinated patients rapidly increased from 111 (95% CI: 41-299) at day 1 to a significantly higher level of 331 at day 3 (95% CI: 202-541), and peaked at 6-10 days with a GMT of 1348 (95% CI: 947-1829) (Fig. 2). According to the random intercept model based on 310 serum samples from 92 unvaccinated patients who provided serial samples throughout, the EV-A71 NAb titre was 40 (95% CI: 9-180) at the day of onset, increased to 402 (95% CI: 317-511) at day 3, and peaked at 2417 on day 13 (95% CI: 1859-3143). In this model, NAb levels maintained above 1240 until 26 months after illness onset (Fig. 3a-b). Kinetics of antibody response was coincident except 2 days earlier reaching peak titre when patients with only one serum sample were added into the model (Supplementary Fig.5).

There was no significant difference between IVIG treatment and non-IVIG treatment group at each time point within 10 days since illness onset. For 182 patients without IVIG treatment, GMTs showed no significant difference among all time groups between 129 mild cases and 53 severe cases (data is not shown).

### 3.4 Sampling time of paired serum samples

92 EV-A71 infected HFMD patients in the dynamic model all presented with fever or rash, 94.6% (87/92) of them developed both symptoms. The average time from the appearance of symptoms until they subsided was 7.2 days (95% CI: 3.0-17.1), this period was defined as the acute phase (Fig. 3c). We calculated the time interval required for a 2, 4 and 8-fold increase in NAb titre, and estimated the sampling time of paired serum samples (Table 2, Supplementary Table 5, Supplementary Table 6). If the first serum sample was collected from EV-A71 infected HFMD patients 0-7 days from symptom onset, we estimated the collection time of the convalescent serum to be 1.7 (95% CI: 1.3-2.3) to 29.3 (95% CI: 20.0-41.3) days after onset to reach a 4-fold rise in antibody titre. Limited by the peak titre at day 13, the acute phase serum should be collected within 4 days of symptom onset. According to the kinetics of the NAb response, therefore, the corresponding convalescent serum should be collected 14.9 days (95% CI: 9.1-23.8) after illness onset. The average time from onset to hospital admission was 2.8 days (95% CI: 0.5-7.0), and from onset to discharge was 10.2 days (95% CI: 5.0-21.0), respectively (Fig. 3d). Therefore, when the acute phase serum was collected within 3 days of illness onset, the convalescent serum could be collected immediately before discharge. In addition, when 2-fold or 8-fold rise in antibody titre was used as the standard, the convalescent serum was estimated to be collected 23.2 (95% CI: 14.4, 36.3) or 26.6 (95% CI: 14.6, 35.3) days after illness onset (Table 2).

**Table 2:** Estimation of sampling time for paired serum samples to reach a 2, 4 and 8-fold increase in NAb titre.

Sampling time of acute phase serum*	Estimated sampling time of convalescent serum (Mean, 95% CI) *		
	2-fold increase	4-fold increase	8-fold increase
0	1.4 (1.0, 1.9)	1.7 (1.3, 2.3)	2.3 (1.8, 2.8)
1	2.3 (2.0, 2.7)	2.9 (2.6, 3.3)	3.6 (3.1, 4.0)
2	3.5 (3.2, 3.9)	4.2 (3.6, 5.1)	5.9 (4.3, 9.5)
3	6.9 (4.8, 10.9)	10.1 (6.3, 17.2)	16.4 (9.9, 26.9)
4	9.3 (6.5, 14.8)	14.9 (9.1, 23.8)	21.4 (12.0, 31.9)
5	11.8 (8.7, 18.4)	24.4 (15.3, 38.3)	22.9 (13.0, 31.6)
6	18.0 (11.6, 28.6)	30.3 (21.2, 42.9)	25.0 (15.0, 33.2)
7	23.2 (14.4, 36.3)	29.3 (20.0, 41.3)	26.6 (14.6, 35.3)

⁎ Days after illness onset.

## 4 Discussion

In this study, we found that EV-A71 infected HFMD patients hospitalised in Henan children's hospital had a strong and persistent NAb response against EV-A71 during hospitalisation and long time after recovery. For serological diagnosis of EV-A71 infection in HFMD patients, when the first serum was collected at 0-7 days of the acute phase, the convalescent serum should be collected 3-4 weeks after illness onset.

Few studies have previously described the kinetics of EV-A71 NAb response in HFMD patients. Our data indicated that the antibody response has already initiated once clinical symptoms appear, and the NAb titre increased with time within a few days after illness onset, similar to what has been reported in previously published studies [13]. The average period between infection and appearance of signs and symptoms in HFMD patients is 3-7 days, meaning that sufficient time has elapsed for many patients to have developed a NAb response [14]. Wang, Y., *at al.* reported that anti-EV-A71 NAb GMT reached the highest level at day 6, then decreased at day 7 and day 8 after disease onset. However, their sample size was small and lacked data from the later sampling times that we present here [15]. Our study showed that EV-A71 NAb GMTs increased to 1348 at 6-10 days, with the estimated peak titre of 2417 on day 13. The differences in peak time and NAb titres obtained by GMT calculation and the model prediction were due to the exclusion of patients with only one serum sample and those were still seronegative two months after illness onset, who presumably did not have EV-A71 infection (see Methods). Our result was higher than that of Yang, C., *at al.* (GMT=79.5) and Nguyet, L. A, *at al.* (GMT=295.1), which might be due to many reasons such as study design, different experimental setup and challenge strains, NAb titres are not always easy to compare between laboratories [13,16]. Nguyet, L. A, *at al.* used B5 sub-genotype EV-A71 strain in the neutralisation test, which was dominant in Vietnam but not the currently prevalent C4a subtype in mainland China. Yang, C., *at al.* used C4b sub-genotype EV-A71 strain, but not all participants had disease caused by EV-A71 infection. In our study, patients’ NAb levels were maintained at a relatively stable and high level (above 1240) until 26 months after the disease, suggesting that natural EV-A71 infection induced an effective and persistent humoral immune response.

Without proven effective therapy for EV-A71 infection, IVIG containing EV-A71 NAb titre above 256 is suggested for clinical treatment of severe HFMD patients in China [10,17]. A meta-analysis of 8 randomised controlled trials showed a benefit of IVIG, with greater effect the higher the dose, at a cost of more adverse events [18]. In our cohort, the median time of hospital admission after illness onset was 3 days, when NAb titres have already reached more than 256 and peaked quickly in the following two weeks. These data indicate that by the time of hospital admission, NAb titres are well established, calling into question the benefit of administering further NAb. However, high-dose IVIG also has both anti-inflammatory effects and adverse effects, meaning the risk/benefit ratio of IVIG for the treatment of HFMD still requires further clarification [19], [20], [21].

In this study, a 4-fold increase in NAb titres within 2 months after onset was not observed in 54.1% (59/109) of patients. Comparing those patients who did not show a 4-fold rise in titre, the first serum samples were collected later (4 days vs. 3 days) and the NAb titres were higher (1448 vs. 362) than those whose titres increased 4-fold or more. Therefore, these patients were presumably sampled when the immune response was already established and too late to be able to show a 4-fold rise. The high antibody titre of the first serum might also be the result of a recent or previous infection, but in the absence of serum samples before illness onset we could not determine this. In addition, 12.8% (14/109) of patients were still seronegative 2 months after the onset. It appeared that, considering factors such as imperfect sensitivity or specificity of the assays and carrier state of the host, detection of virus in a sample does not equal to setting up an efficient infection. A small fraction of people may fail to produce a measurable level of NAb because the response is directed to non-neutralising epitopes, or because the response itself is absent or low level, due to immune insufficiency or other unknown factors [22]. There was no clinical suggestion of immune deficiency in these patients that we studied here.

A 4-fold rise in EV-specific NAb titre between acute and convalescent phases is diagnostic of recent infection [7]. However, previous studies have not clearly defined the sampling time of paired sera, which still needs support from experimental evidence [7,10]. According to our results, if a 4-fold increase was used as the criterion for serological diagnosis, and considering the feasibility of clinical sampling, the acute phase serum should be collected within 3 days of illness onset, then the corresponding convalescent serum could be collected before discharge. Regardless of the discharge time, the acute phase serum should be collected within 4 days (the earlier the better), and the corresponding convalescent serum should be collected 15 days after illness onset.

A 2-fold rise is usually not considered sufficient evidence of infection because of inherent measurement errors. Studies on influenza infection suggest that demonstrating a 4-fold rise is necessary to make a specific diagnosis for individual cases, but a lower fold increase may be sufficient if the objective is to estimate population attack rates [23]. Limited by the peak NAb titre and the early collection time of the first serum sample to reliably detect an increase of this magnitude, it is reasonable to use a 4-fold rise in NAb titres as the standard for serological diagnosis.

Our analysis and interpretation of the findings is subject to several limitations. First, a large proportion of participants were lost to follow up after discharge, which might cause deviation in the estimation of the antibody response. Sparse data on antibody titres might affect the estimation of the sampling time of paired serum samples, leading to a shorter time interval required for an 8-fold increase in NAb titre than a 4-fold increase, when the acute phase serum was collected 5 days or later after illness onset. Secondly, the patients who participated in the follow-up after discharge were more severely ill and received IVIG treatment, the effect on the predicted kinetics of the antibody response cannot be excluded because of lacking power to make a robust comparison due to the small sample size. Thirdly, this is a single-centre localised study, where participants were recruited three years ago, and the influence of age, clinical treatment and other factors were not fully considered, therefore the extrapolation of the results might be limited.

To our knowledge, this study represents the first attempt to construct a kinetic model of the NAb response to EV-A71 over time in HFMD patients using data from serum samples at multiple time points during 2 years after illness onset. It is also one of the largest studies of its type addressing this important question. We found that strong NAb responses were elicited by natural EV-A71 infection, and NAb titres were maintained at a high level until 2 years after illness onset. In addition, we recommend the sampling time for paired serum samples, that the acute phase sample should be taken as early as possible, certainly within 3-4 days after illness onset, providing a firm basis for the optimization of serological diagnosis of EV-A71 infection in HFMD patients.

## Declaration of Competing Interest

H Yu has received research funding from Sanofi Pasteur, GlaxoSmithKline, Yichang HEC Changjiang Pharmaceutical Company, and Shanghai Roche Pharmaceutical Company, outside this study. All authors declare no competing interests.

## Appendix Supplementary materials

Image, application 1

Image, application 2

Image, application 3
