# Supplementary material for: Kinetics of the neutralising antibody response in patients with hand, foot, and mouth disease caused by EV-A71: A longitudinal cohort study in Zhengzhou during 2017-2019
Source: eBioMedicine. 2021 May 25;68:103398. doi: 10.1016/j.ebiom.2021.103398 (PMC8170117; doi:10.1016/j.ebiom.2021.103398)
Supplement: Supplementary file 2 [file mmc2.docx]

# Supplementary Tables

**Supplementary Table 1**

The number of patients and samples available during hospitalisation and follow-up period.

| Time points of sampling | All 264 patients | |  | Patients receiving EV-A71 vaccination | |  | Patients receiving IVIG treatment ^b^ | |
| --- | --- | --- | --- | --- | --- | --- | --- | --- |
|  | No. of  patients | No. of  samples |  | No. of  patients | No. of  samples |  | No. of  patients | No. of  samples |
| During hospitalisation ^a^ | 262 | 349 |  | 12 | 17 |  | 72 | 142 |
| 2-week follow-up | 73 | 73 |  | 6 | 6 |  | 33 | 33 |
| 3-month follow-up | 32 | 32 |  | 3 | 3 |  | 20 | 20 |
| 6-month follow-up | 44 | 44 |  | 3 | 3 |  | 25 | 25 |
| 1.5-month follow-up | 26 | 26 |  | 2 | 2 |  | 16 | 16 |

^a^ Each patient had one or more serum samples collected during hospitalisation (at admission, disease progress, and discharge), except for 2 patients only had serum samples collected during follow-up period.

^b^ The median time of commencing IVIG administration was 3 days (IQR: 2-4), and the treatment finished at a median of 5 days (IQR: 4-6) from illness onset. A total of 79 serum samples were collected during IVIG treatment.

**Supplementary Table 2**

Details of 524 serum samples collected from 264 patients.

|  | No. of serum samples collected from each participant | | | | | | |
| --- | --- | --- | --- | --- | --- | --- | --- |
|  | 1 | 2 | 3 | 4 | 5 | 6 | 7 |
| No. (%) of participants | 150 (56.8) | 43 (16.3) | 27 (10.2) | 21 (8.0) | 17 (6.4) | 4 (1.5) | 2 (0.8) |
| No. (%) of serum samples | 150 (28.6) | 86 (16.4) | 81 (15.5) | 84 (16.0) | 85 (16.2) | 24 (4.6) | 14 (2.7) |

**Supplementary Table 3**

Baseline characteristics of patients being vaccinated against EV-A71 or not

| Characteristics | Vaccinated patients (n=12) | Unvaccinated patients (n=252) | P-value |
| --- | --- | --- | --- |
| Sex |  |  | 0.222 |
| Male | 5 (41.7) | 158 (62.7) |  |
| Female | 7 (58.3) | 94 (37.3) |  |
| Age, years |  |  | 0.176 |
| <2 | 8 (66.7) | 107 (42.5) |  |
| ≥2 | 4 (33.3) | 145 (57.5) |  |
| Median (IQR) time (days) from symptom onset to hospital admission | 2 (2, 4) | 2 (2, 4) | 0.465 |
| Median (IQR) time (days) of LOS | 5 (4, 5) | 5 (4, 6) | 0.598 |
| Clinical severity |  |  | 0.075 |
| Mild | 9 (75.0) | 112 (44.4) |  |
| Severe | 3 (25.0) | 140 (55.6) |  |

Data are no. (%) of patients, unless otherwise indicated.

**Supplementary Table 4**

Baseline characteristics of HFMD patients being invited or participated in the follow-up or not.

| Characteristics | All EV-A71 RNA test-positive patients (N=264) | | |  | Patients invited for follow-up (N=199) | | |
| --- | --- | --- | --- | --- | --- | --- | --- |
|  | Invited for  follow-up (n=199) | Excluded for follow-up (n=65) | P-value |  | Participated  (n=83) | Refused  (n=116) | P-value |
| Sex |  |  | 1.000 |  |  |  | 0.953 |
| Male | 123 (61.8) | 40 (61.5) |  |  | 52 (62.7) | 71 (61.2) |  |
| Female | 76 (38.2) | 25 (38.5) |  |  | 31 (37.3) | 45 (38.8) |  |
| Age, years |  |  | 0.814 |  |  |  | 0.818 |
| < 2 | 88 (44.2) | 27 (41.5) |  |  | 38 (45.8) | 50 (43.1) |  |
| ≥ 2 | 111 (55.8) | 38 (58.5) |  |  | 45 (54.2) | 66 (56.9) |  |
| EV-A71 vaccination |  |  | 0.304 |  |  |  | 0.531 |
| Yes | 11 (5.5) | 1 (1.5) |  |  | 6 (7.2) | 5 (4.3) |  |
| No | 188 (94.5) | 64 (98.5) |  |  | 77 (92.8) | 111 (95.7) |  |
| Median (IQR) time (days) from symptom onset to hospital admission | 3 (2, 4) | 3 (2, 3) | 0.196 |  | 3 (2, 4) | 3 (2, 4) | 0.530 |
| Median (IQR) time (days) of LOS | 5 (4, 7) | 5 (4, 5) | 0.001 |  | 6 (5, 8) | 5 (4, 6) | <0.001 |
| Clinical severity |  |  | 0.001 |  |  |  | 0.058 |
| Mild | 79 (39.7) | 42 (64.6) |  |  | 26 (31.3) | 53 (45.7) |  |
| Severe | 120 (60.3) | 23 (35.4) |  |  | 57 (68.7) | 63 (54.3) |  |

Data are no. (%) of patients, unless otherwise indicated. IQR=interquartile range. LOS=length of hospital stay.

**Supplementary Table 5**

Comparison of the estimated GMTs during the acute phase using different imputation methods.

| Acute phase  (days since  illness onset) | Baseline analysis:  GMTs (95% CI) predicted by GLM | Sensitivity analysis 1:  GMTs (95% CI) calculated at each time point | Sensitivity analysis 2:  GMTs (95% CI) predicted by linear mixed model |
| --- | --- | --- | --- |
| 1 | 103 (46, 231) | 133 (44, 404) | 403 (269, 603) |
| 2 | 222 (154, 320) | 138 (73, 261) | 494 (346, 705) |
| 3 | 402 (317, 511) | 331 (200, 547) | 606 (444, 827) |
| 4 | 610 (481, 774) | 585 (389, 881) | 743 (565, 977) |
| 5 | 830 (673, 1023) | 577 (298, 1120) | 911 (712, 1165) |
| 6 | 1086 (889, 1326) | 1065 (673, 1686) | 1117 (886, 1408) |
| 7 | 1367 (1114, 1679) | 1185 (376, 3738) | 1370 (1086, 1727) |

**Supplementary Table 6**

Comparison of the estimated sampling time of convalescent serum samples.

| Sampling time  for acute phase sample  (days since illness onset) | Estimated sampling time for convalescent sample^*^ (Mean, 95% CI) | | | | | | | | |
| --- | --- | --- | --- | --- | --- | --- | --- | --- | --- |
|  | 2-fold increase | | | 4-fold increase | | | 8-fold increase | | |
|  | Baseline  analysis | Sensitivity  analysis 1 | Sensitivity  analysis 2 | Baseline  analysis | Sensitivity  analysis 1 | Sensitivity  analysis 2 | Baseline  analysis | Sensitivity  analysis 1 | Sensitivity  analysis 2 |
| 1 | 2.3 (2.0, 2.7) | 2.6 (2.3, 3.1) | 3.4 (3.0, 3.9) | 2.9 (2.6, 3.3) | 3.1 (2.7, 3.6) | 4.7 (3.9, 5.9) | 3.6 (3.1, 4.0) | 3.9 (3.4, 4.4) | 13.0 (8.1, 21.2) |
| 2 | 3.5 (3.2, 3.9) | 3.4 (3.1, 3.8) | 3.9 (3.5, 4.3) | 4.2 (3.6, 5.1) | 3.9 (3.3, 4.7) | 4.9 (4.1, 6.1) | 5.9 (4.3, 9.5) | 5.3 (3.8, 8.5) | 13.4 (8.2, 21.4) |
| 3 | 6.9 (4.8, 10.9) | 6.7 (4.6, 10.6) | 7.1 (4.9, 11.1) | 10.1 (6.3, 17.2) | 9.4 (5.8, 15.8) | 12.0 (7.4, 20.0) | 16.4 (9.9, 26.9) | 11.8 (6.6, 19.4) | 20.4 (13.0, 31.3) |
| 4 | 9.3 (6.5, 14.8) | 9.3 (6.5, 14.8) | 10.6 (7.4, 16.5) | 14.9 (9.1, 23.8) | 14.9 (9.1, 23.8) | 20.0 (11.8, 32.5) | 21.4 (12.0, 31.9) | 21.4 (12.0, 31.9) | 19.7 (11.1, 28.5) |
| 5 | 11.8 (8.7, 18.4) | 12.7 (8.2, 20.8) | 11.8 (8.7, 18.4) | 24.4 (15.3, 38.3) | 17.8 (11.2, 27.2) | 24.4 (15.3, 38.3) | 22.9 (13.0, 31.6) | 22.6 (13.9, 31.4) | 22.9 (13.0, 31.6) |
| 6 | 18.0 (11.6, 28.6) | 18.0 (11.6, 28.6) | 18.0 (11.6, 28.6) | 30.3 (21.2, 42.9) | 30.3 (21.2, 42.9) | 30.3 (21.2, 42.9) | 25.0 (15.0, 33.2) | 25.0 (15.0, 33.2) | 25.0 (15.0, 33.2) |
| 7 | 23.2 (14.4, 36.3) | 23.2 (14.4, 36.3) | 23.2 (14.4, 36.3) | 29.3 (20.0, 41.3) | 29.3 (20.0, 41.3) | 29.3 (20.0, 41.3) | 26.6 (14.6, 35.3) | 26.6 (14.6, 35.3) | 26.6 (14.6, 35.3) |

^*^ Baseline analysis is based on GMTs (95% CI) of acute phase serum predicted by the generalised linear mixed (GLM) model in the original manuscript, Sensitivity analysis 1 is based on that calculated at each time point in the acute phase, Sensitivity analysis 2 is based on that predicted by linear mixed model (Supplementary Table 5).

Supplementary Figures**
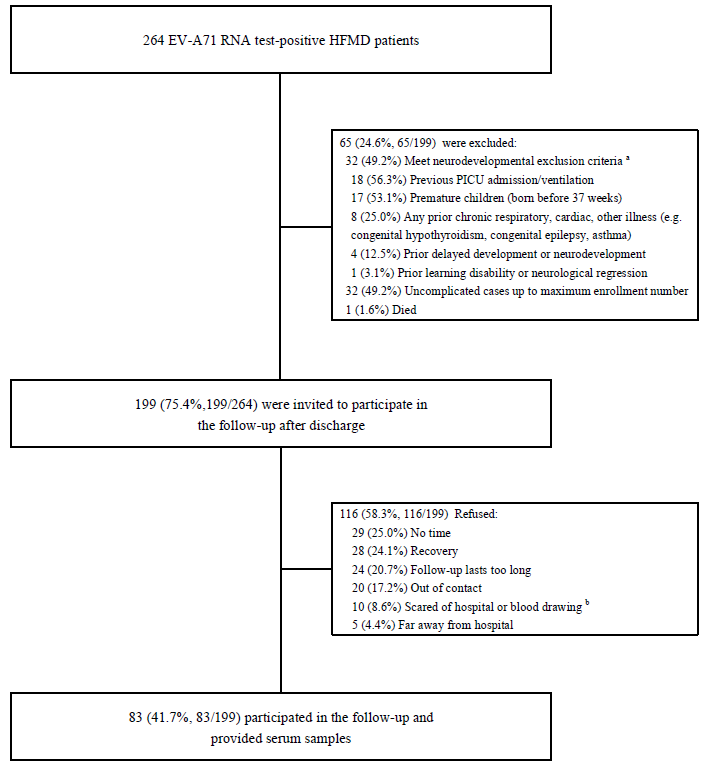
**

**Supplementary Fig. 1.** Flow chart of participants enrolment for follow-up.

^a^ One patient could meet one or more criteria.

^b^ 7 of 10 patients participated in the follow-up but declined to provide serum samples.

**
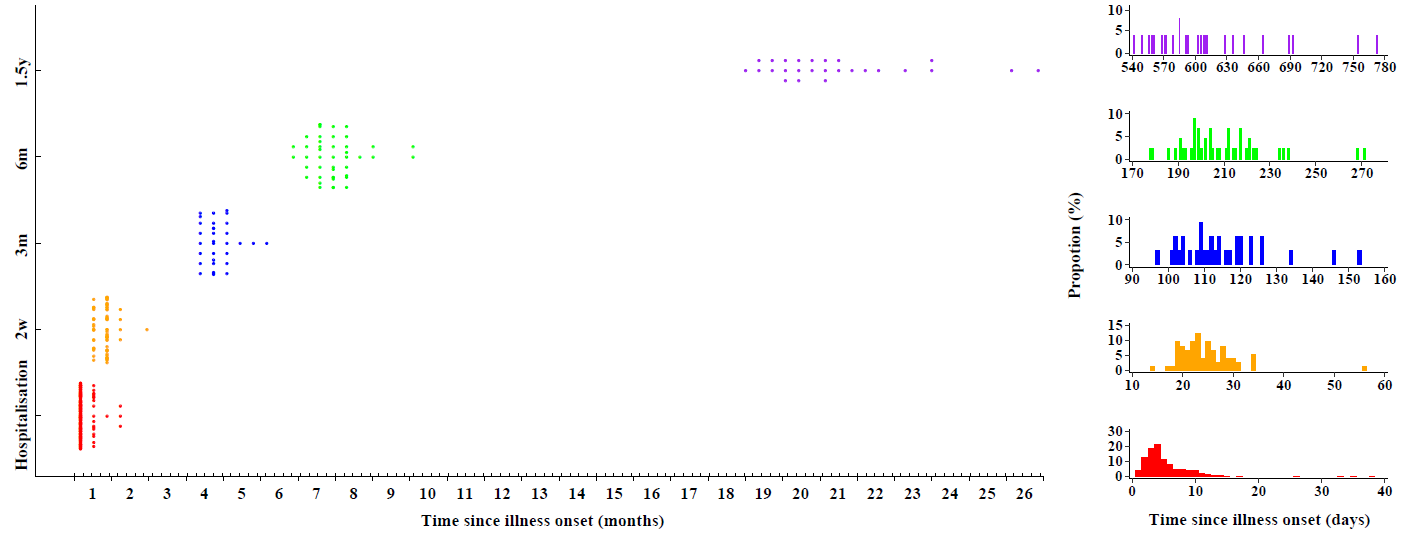
**

**Supplementary Fig. 2.** Sampling time of serum samples after illness onset. One dot represents a serum sampling. Red, orange, blue, green, and purple represent sample collection during hospitalisation, 2-week, 3-month, 6-month and 1.5-year follow-up, corresponding median (range) sampling time are 5 days (0-38 days), 23 days (14-56 days), 113 days (97-153 days), 205 days (178-271 days), 598 days (542-773 days) after illness onset, respectively. Right panel shows the distribution of sample size during each period.

**
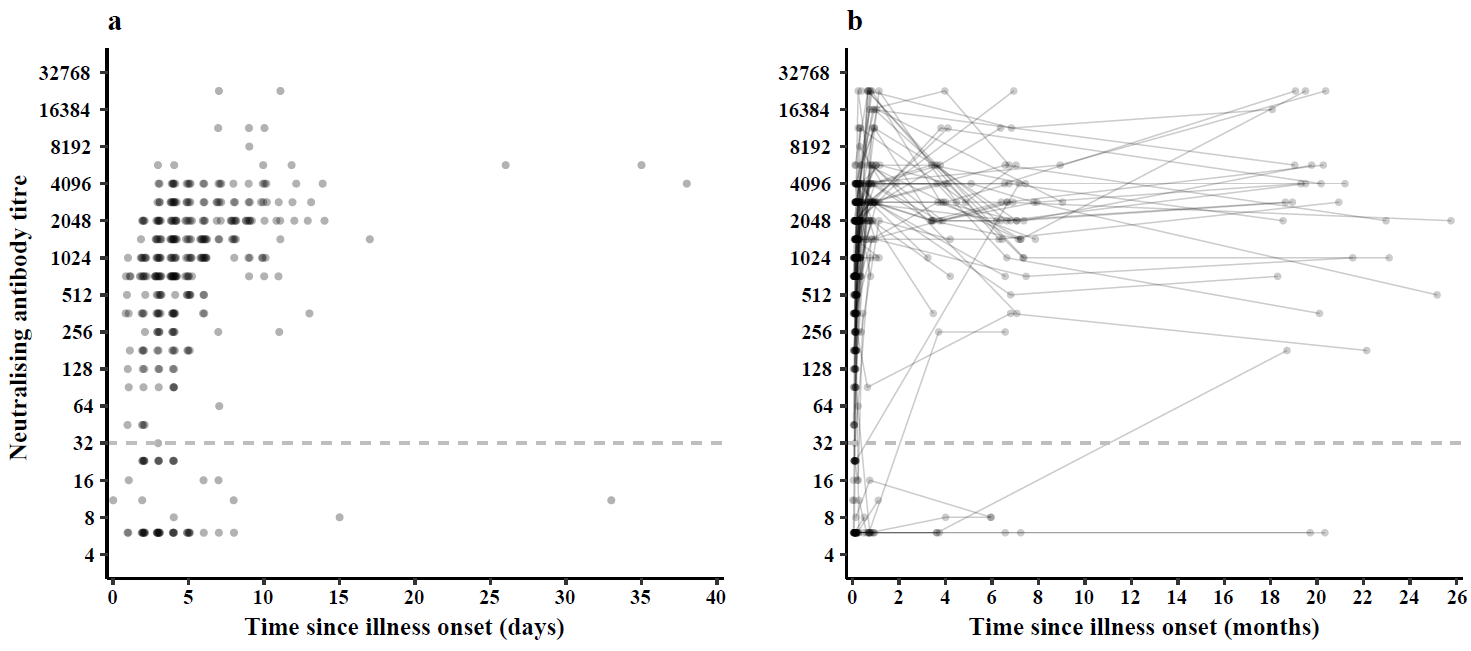
**

**Supplementary Fig. 3.** EV-A71 neutralising antibody titres at different times after illness onset for each patient. **(a)** during 0-40 days after illness onset; **(b)** during whole study period (0-26 months after illness onset). Dots show antibody titre for each sample, each combination of dot represents an individual, points from the same patient are connected. Gray dotted line indicates threshold for positive titre (≥32).


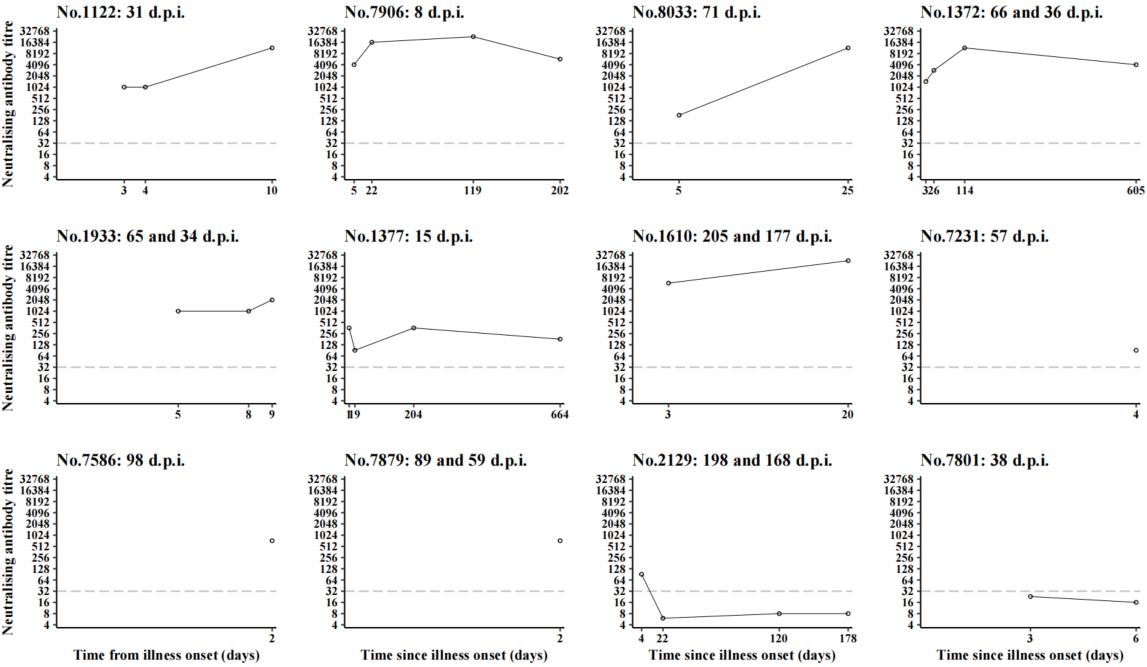


**Supplementary Fig. 4.** Antibody response of each patient who received EV-A71 vaccination before illness onset. Title of each panel including the ID No. of the patient and days post-immunization (d.p.i.) before their illness onset. Five patients (No.1372, 1933, 1610, 7879, 2129) received two doses of the vaccine, the others received only one dose.


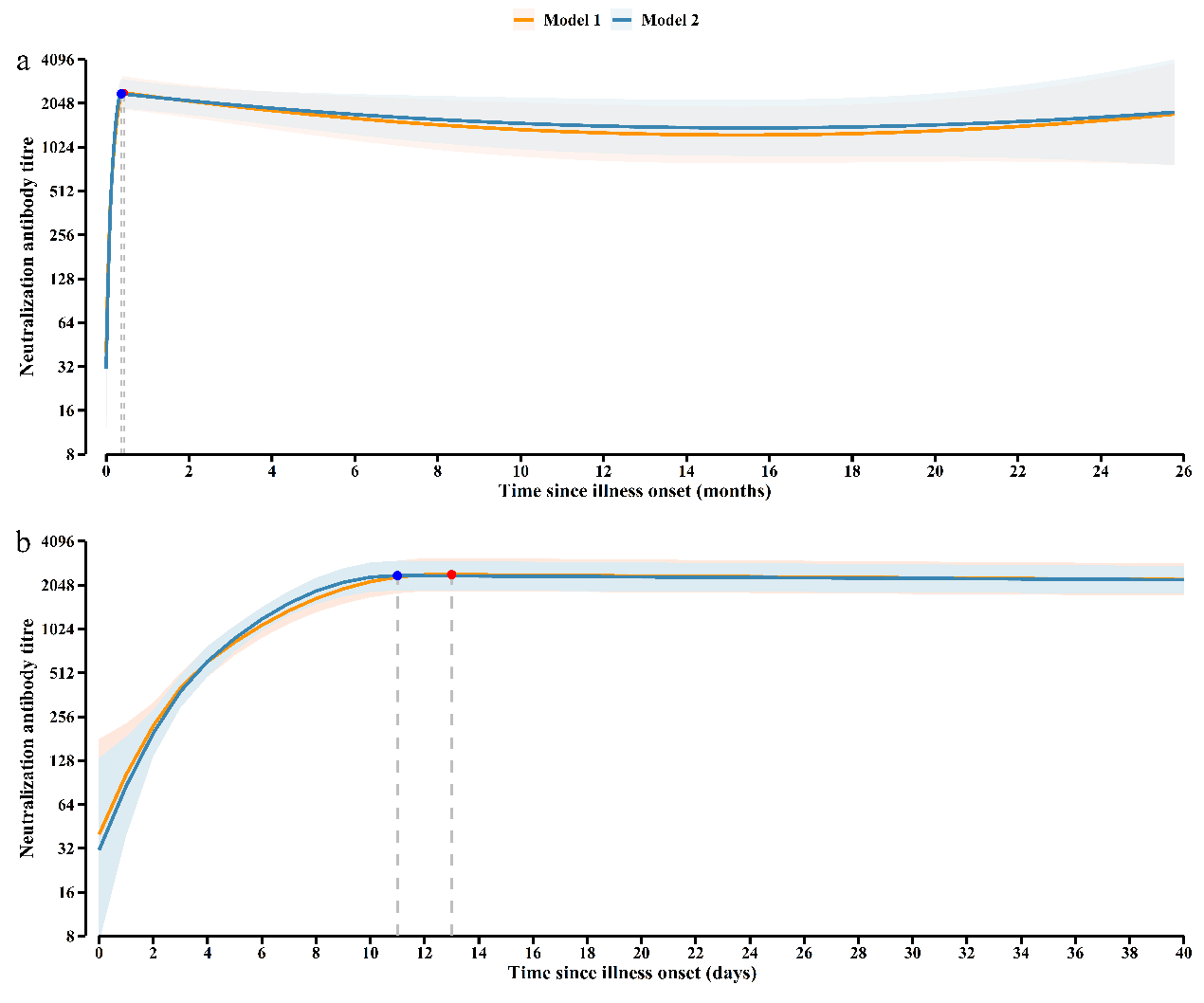


**Supplementary Fig. 5.** Two models of EV-A71 neutralising antibody responses over time after illness onset. **(a)** during whole study period (0-26 months after illness onset); **(b)** during 0-40 days after illness onset. Model 1 (orange) include 310 serum samples from 92 unvaccinated patients who provided serial samples throughout, model 2 (blue) add 147 unvaccinated patients with only one serum sample. The curves and ribbons represent average NAb titre and 95% CI. The dots indicate the peak titre and corresponding sampling time.
