# Supplementary material for: Kinetics of the neutralising antibody response in patients with hand, foot, and mouth disease caused by EV-A71: A longitudinal cohort study in Zhengzhou during 2017-2019
Source: eBioMedicine. 2021 May 25;68:103398. doi: 10.1016/j.ebiom.2021.103398 (PMC8170117; doi:10.1016/j.ebiom.2021.103398)
Supplement: Supplementary file 3 [file mmc3.docx]

**Supplementary Table 1**

The number of patients and samples available during hospitalisation and follow-up period.

**Supplementary Table 2**

Details of 524 serum samples collected from 264 patients.

**Supplementary Table 3**

Baseline characteristics of patients being vaccinated against EV-A71 or not

**Supplementary Table 4**

Baseline characteristics of HFMD patients being invited or participated in the follow-up or not.

**Supplementary Table 5**

Comparison of the estimated GMTs during the acute phase using different imputation methods.

**Supplementary Table 6**

Comparison of the estimated sampling time of convalescent serum samples.

**Supplementary Fig. 1.** Flow chart of participants enrolment for follow-up.

**Supplementary Fig. 2.** Sampling time of serum samples after illness onset.

**Supplementary Fig. 3.** EV-A71 neutralising antibody titres at different times after illness onset for each patient.

**Supplementary Fig. 4.** Antibody responses of 12 patients who received EV-A71 vaccination before illness onset.

**Supplementary Fig. 5.** Two models of EV-A71 neutralising antibody responses over time after illness onset.
